# Interaction between corticotropin-releasing factor, orexin, and dynorphin in the infralimbic cortex may mediate exacerbated alcohol-seeking behavior

**DOI:** 10.1016/j.ynstr.2024.100695

**Published:** 2024-11-19

**Authors:** Francisco J. Flores-Ramirez, Jessica M. Illenberger, Rémi Martin-Fardon

**Affiliations:** a Department of Molecular Medicine, The Scripps Research Institute, La Jolla, CA, USA; b Department of Psychology, California State University, San Marcos, CA, USA

## Abstract

A major challenge for the treatment of alcohol use disorder (AUD) is relapse to alcohol use, even after protracted periods of self-imposed abstinence. Stress significantly contributes to the chronic relapsing nature of AUD, given its long-lasting ability to elicit intense craving and precipitate relapse. As individuals transition to alcohol dependence, compensatory allostatic mechanisms result in insults to hypothalamic-pituitary-adrenal axis function, mediated by corticotropin-releasing factor (CRF), which is subsequently hypothesized to alter brain reward pathways, influence affect, elicit craving, and ultimately perpetuate problematic drinking and relapse vulnerability. Orexin (OX; also called hypocretin) plays a well-established role in regulating diverse physiological processes, including stress, and has been shown to interact with CRF. Interestingly, most hypothalamic cells that express *Ox* mRNA also express *Pdyn* mRNA. Both dynorphin and OX are located in the same synaptic vesicles, and they are co-released. The infralimbic cortex (IL) of the medial prefrontal cortex (mPFC) has emerged as being directly involved in the compulsive nature of alcohol consumption during dependence. The IL is a CRF-rich region that receives OX projections from the hypothalamus and where OX receptor mRNA has been detected. Although not thoroughly understood, anatomical and behavioral pharmacology data suggest that CRF, OX, and dynorphin may interact, particularly in the IL, and that functional interactions between these three systems in the IL may be critical for the etiology and pervasiveness of compulsive alcohol seeking in dependent subjects that may render them vulnerable to relapse. The present review presents evidence of the role of the IL in AUD and discusses functional interactions between CRF, OX, and dynorphin in this structure and how they are related to exacerbated alcohol drinking and seeking.

## 1 Overview

**Fig. 1:**
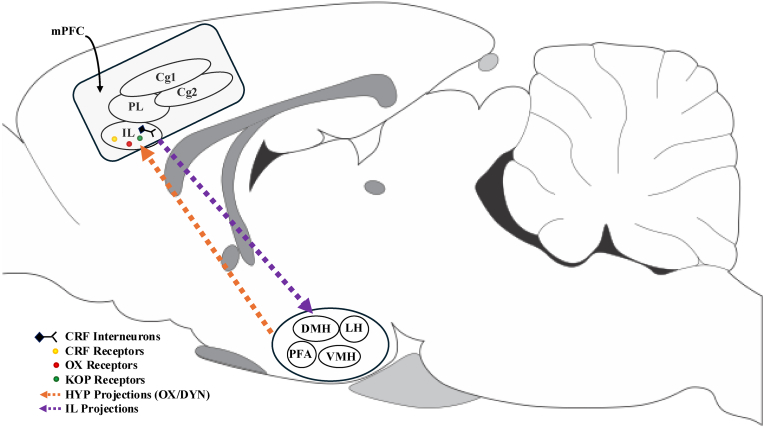
Schematic diagram of HYP projections to the infralimbic cortex (orange arrows), and IL projection back to the HYP (purple arrow). Locations of putative CRF-expressing interneurons, CRF receptors (yellow circles), OX receptors (red circles), and KOP receptors (green circles). DMH, dorsomedial hypothalamus; IL, infralimbic cortex; LH, lateral hypothalamus; PFA, perifornical area; VMH, ventromedial hypothalamus; mPFC, medial prefrontal cortex; Cg1, cingulate cortex 1; Cg2, cingulate cortex 2; PL, prelimbic cortex; CRF, corticotropin-releasing factor; KOP, kappa opioid; OX, orexin; DYN, dynorphin. Modified from: Cahill et al. (2022); Kim and Martin-Fardon (2020); Moorman et al. (2015); Paxinos and Watson (1986); Swanson et al. (1983); Tsujino and Sakurai (2013); Vertes (2004). (For interpretation of the references to colour in this figure legend, the reader is referred to the Web version of this article.)

This review will delve into the complex interactions between corticotropin-releasing factor (CRF), hypocretin/orexin (OX), and dynorphin (DYN) within the infralimbic cortex (IL) and their significant roles in modulating exacerbated alcohol-seeking behavior and relapse. It will highlight the corticotropin releasing factor (CRF), a key player in the stress response, and its influence on the hypothalamic-pituitary-adrenal (HPA) axis to contribute to the compulsive nature of alcohol use disorder (AUD). In this context, compulsivity is operationally defined as the uncontrollable urge to consume alcohol, even when it causes physical, psychological, or social harm, and that is often accompanied by repeated unsuccessful attempts to reduce or stop drinking. This manuscript will underscore the importance of the IL, a region rich in CRF receptors and receiving significant OX projections, in the etiology and pervasiveness of AUD. It discusses how the co-release of OX and DYN within the IL can influence neuronal activity and behavioral outcomes, and by examining the functional dynamics of these neuropeptides (see Fig. 1) this manuscript aims to provide insights into potential therapeutic targets for preventing relapse and managing AUD. The interactions between these systems are complex and involve reciprocal feedback mechanisms that can either promote or inhibit alcohol-seeking behavior; thus, understanding these interactions could lead to improved clinical outcomes that aim to address the underlying neurobiological mechanisms of AUD. Ultimately, this comprehensive review will highlight the need for further research to explore these pathways and their implications for treatment strategies.

## 2 Background

Alcohol use disorder is one of the most prevalent mental health afflictions, representing a major public health, economic, and social concern, given its contribution to disability and preventable death worldwide (Grant et al., 2015; Grant et al., 2004; MacKillop et al., 2022; Rehm et al., 2015; Witkiewitz et al., 2019; Yaseen et al., 2024). Although there have been major developments in the treatment and management of several chronic health conditions such as metabolic disorders, cardiovascular disease, and autoimmune conditions, safe and effective treatments for individuals who suffer from AUD remain elusive in the clinical setting (Pierce, O'Brien, Kenny and Vanderschuren, 2012; Yaseen et al., 2024). A major challenge that clinicians encounter when treating AUD is the prevalence of relapse to alcohol use, even after protracted periods of forced or self-imposed abstinence. Stress, defined as any stimulus that disturbs physiological homeostasis, is a major factor that has long been thought to significantly contribute to the chronic relapsing and compulsive nature of alcohol dependence, given its long-lasting ability to elicit craving in individuals who are recovering from AUD and its ability to reinstate alcohol-seeking behavior (Sinha, 2024; Stephens and Wand, 2012). Several studies have found that people who experience heightened social stressors exhibit higher rates of relapse following treatment for AUD than those who do not experience stress (S. A. Brown et al., 1990; Noone et al., 1999; Sinha, 2024).

### 2.1 Role of stress in alcohol-seeking behavior

Although the exact mechanisms by which stress underlies the vulnerability to AUD and the higher likelihood of alcohol relapse are not thoroughly understood, the HPA axis has long been thought to play a significant role and has been studied extensively in past decades (Wand, 2008). Acutely, and at low doses, alcohol has an array of effects on the human body, to include stimulation, euphoria, anxiolysis, and sedation (Knight et al., 2020). Paradoxically, recurring cycles of alcohol use have been suggested to significantly dysregulate responsivity to stress that is mediated by the HPA axis. Thus, as individuals transition to alcohol dependence, compensatory allostatic mechanisms result in insults to HPA axis function, which is subsequently hypothesized to alter brain reward pathways, influence affect, elicit craving, and ultimately perpetuate problematic drinking and relapse vulnerability (Becker and Mulholland, 2014; Koob and Le Moal, 2001; Stephens and Wand, 2012). Long-lasting adaptations of stress systems underscore the transition from controlled drinking, which is thought to be primarily motivated by positive reinforcement processes, to uncontrolled drinking, which is largely driven by negative affect and negative reinforcement processes. The most prominent hallmark of this “dark side” of addiction is the dysregulation of neurophysiological systems that are involved in reward and stress and that are continuously pushed beyond a normal homeostatic set point. These neuroplastic changes then underlie the compulsive nature of alcohol seeking and drinking during dependence and the vulnerability to relapse (Koob, 2009; Koob and Le Moal, 1997)

### 2.2 Corticotropin-releasing factor is critical in the mediation of alcohol-seeking behavior

Corticotropin-releasing factor is a 41-amino-acid residue single-chain polypeptide that was first identified in 1981 to play a central role in regulating immune, endocrine, autonomic, and particularly neural responsivity to stress (Vale et al., 1981). Functioning as a neuromodulator, the CRF-binding protein (CRF-BP), a glycoprotein, regulates the extracellular availability of CRF for potential receptor binding (Behan et al., 1995). CRF exerts its endocrine and neurobehavioral effects by binding to two G-protein-coupled receptors, CRF_1_ and CRF_2_. CRF has been shown to have 10-fold greater affinity for CRF_1_ receptors *vs*. CRF_2_ receptors. The activation of both receptor subtypes results in the activation of cyclic adenosine monophosphate second messenger pathways (M. Perrin et al., 1995; M. H. Perrin and Vale, 1999). Interestingly, both receptor subtypes can be differentially found throughout the brain, with many overlapping regions, although CRF_1_ receptors are more ubiquitous (Van Pett et al., 2000). Hypothalamic CRF drives the HPA axis via CRF_1_ receptor activation in the anterior pituitary, which stimulates adrenocorticotropic hormone (ACTH) secretion, ultimately leading to glucocorticoid production and release from the adrenal gland (Vale et al., 1981). Considering that stress is implicated in the development of dysregulated, compulsive alcohol use, extensive research has focused on uncovering CRF's involvement in the etiology and pervasiveness of AUD (Quadros et al., 2016).

Notably, exposure to alcohol itself may act as an acute stressor, triggering activation of the HPA axis, an effect that has been observed in humans who voluntarily drink alcohol (Ekman et al., 1994; Lex et al., 1991). At the preclinical level, the stress-inducing nature of alcohol exposure has also been demonstrated in experimental approaches that feature either forced (Rivier, 1993; Rivier et al., 1984) or voluntary (Richardson, Lee, O'Dell, Koob and Rivier, 2008) alcohol administration. A significant positive correlation was found between blood alcohol levels in male rats after intraperitoneal alcohol administration and various measures of hormonal stress responsivity, in which incremental doses of alcohol (from 0.5 to 4.0 g/kg) significantly and dose-dependently increased plasma corticosterone levels (Ellis, 1966). Interestingly, this alcohol-induced hypersecretion of ACTH does not change according to the mode of alcohol administration—both acute intraperitoneal and intragastric alcohol administration dose-dependently increased ACTH, and blood levels of alcohol and ACTH were strongly correlated, regardless of administration route (Ogilvie et al., 1997). Altogether, these studies demonstrate that the transition from casual, controlled alcohol use to alcohol dependence may be explained at least partially by long-lasting adaptations of stress pathways that arise as a function of stress responsivity to acute alcohol exposure, which ultimately impairs HPA axis reactivity, ultimately resulting in negative affect and alcohol craving that exacerbating the propensity to alcohol relapse during self-imposed abstinence (Heilig and Koob, 2007; Koob, 2009; Stephens and Wand, 2012).

A large body of literature demonstrates that CRF_1_ receptor antagonism results in a significant reduction of alcohol intake, and this effect was shown to be more pronounced when the motivation for alcohol was higher. Subcutaneous administration of the CRF_1_ receptor antagonist *N*,*N*-bis(2-methoxyethyl)-3-(4-methoxy-2-methylphenyl)-2,5-dimethyl-pyrazolo[1,5-*a*]pyrimidin-7-amine (MPZP) significantly decreased dependence-induced exacerbations of alcohol drinking in alcohol-preferring (P) male rats, an effect that was not observed in their nondependent counterparts (Gilpin et al., 2008). Similarly, decreases in home-cage voluntary drinking and operant self-administration were observed in alcohol-dependent but not nondependent male C57BL/6J mice and rats male Wistar, alcohol-preferring P, and Sardinian alcohol-preferring (sP) rats that were administered with other CRF_1_ receptor antagonists, such as [D-Phe^12^,Nle^21,38^,Cα MeLeu^37^]-rCRF_(12–41)_ (D-Phe-CRF_12–41_), *N*-ethyl-4-[4-3fluorophenyl)-3,6-dihydro-2*H*-pyridin-1-yl]-6-methyl-*N*-(2-methylsulfanyl-4-propan-2-ylphenyl)pyrimidin-2-amine (CRA1000), *N*-butyl-*N*-ethyl-2,5-dimethyl-7-(2,4,6-trimethylphenyl)-7*H*-pyrrolo[3,2-*e*]pyrimidin-4-amine (CP154,526), and 4-(*N*-ethyl-n-4-hydroxybutyl)amino-2,5,6-trimethyl-7-92,4,6-trimethylphenyl)pyrrolo[2,4-*d*]pyrimidine hydrochloride (LWH-63), systemically or in the central nucleus of the amygdala (CeA; (Finn et al., 2007; Funk, O'Dell, Crawford and Koob, 2006; Overstreet et al., 2007; Sabino et al., 2006). Moreover, CP154,526, *N*-butyl-*N*-ethyl-[2,5,6-trimethyl-7-(2,4,6-trimethylphenyl)-7*H*-pyrrolo[2,3-d]pyrimidin-4-yl]amino-1-butanol (antalarmin) and 3,6-dimethyl-*N*-pentan-3-yl-2-(2,4,6-trimethylphenoxy)pyridin-4-amine (CP376395) significantly reduced both binge-like and stress-induced increases in alcohol drinking in male BALB/cJ and C57BL/6J mice and male Long-Evans rats (Lowery et al., 2008; Lowery-Gionta et al., 2012; Simms et al., 2014). Notably, however, some studies reported that stress-induced alterations of alcohol drinking were not prevented by pretreatment with the CRF_1_ receptor antagonist 3-[6-(dimethylamino)-4-methyl-3-pyridinyl]-2,5-dimethyl-*N*,*N*-dipropylpyrazolo[1,5-*a*]pyrimidin-7-amine (R121919), and CP154,526 reduced alcohol drinking in both male C57BL/6J mice and male Long-Evans rats that were subjected to both high and low intake conditions (Hwa et al., 2013; Yang et al., 2008). More recently, our group showed that CRF_1_ receptor antagonism with CP154,526 prevented the footshock stress-induced reinstatement of alcohol-seeking behavior in nondependent male Wistar rats (Flores-Ramirez et al., 2022a, Flores-Ramirez et al., 2022b). Lastly, administration of the selective CRF_2_ receptor agonist urocortin 3 (Ucn3) reduced 10% alcohol solution consumption using a 2-h limited-access approach and binge-like alcohol drinking in male C57BL/6J mice (Lowery et al., 2010; Sharpe and Phillips, 2009). Altogether, this literature supports the notion that post-dependent states that are marked by negative emotionality and a loss of control over alcohol intake are mediated at least partially by CRF receptor signaling, with CRF_1_ receptors perhaps playing a more significant role than CRF_2_ receptors.

## 3 Infralimbic cortex plays a pivotal role in extinction and reinstatement of conditioned fear and drug responding

The medial prefrontal cortex (mPFC) processes and integrates new information about habitual behavior to modify this behavior (Kalivas, 2008). The activation of two subregions of the mPFC (i.e., prelimbic cortex [PL] and IL) helps determine behavioral responses in conditioned contexts. The IL and PL have opposing influences (Killcross and Coutureau, 2003) over conditioned fear responding (Sierra-Mercado et al., 2006) and drug seeking (McFarland et al., 2004), particularly during extinction training and reinstatement testing. Of course, the role of the IL in the extinction of fear conditioning was established before establishing its role in the extinction of drug-seeking behavior.

### 3.1 Infralimbic cortex, fear, and stress

Fear and stress activate the CeA and then the ventral tegmental area (VTA), which elicits dopamine release in the PL to promote learned behaviors, such as conditioned fear (McFarland et al., 2004). In contrast, IL activity increases during acquisition and expression of the extinction of conditioned fear (Knapska and Maren, 2009; Milad and Quirk, 2002; Millan et al., 2011). Likewise, in the extinction setting, neurons in the IL respond to stimuli that are associated with conditioned fear to reduce conditioned fear responses (Milad and Quirk, 2002). Stimulating the IL during extinction trials decreased conditioned fear responding and improved extinction recall 24 h later (Milad et al., 2004). The IL projects directly to the nucleus accumbens (NAC) shell (Sesack et al., 1989) and it was shown that inactivation of the IL (Quirk et al., 2000; Sierra-Mercado et al., 2006) or the NAC shell impairs extinction performance (Peters et al., 2008). The plasticity of glutamatergic signaling in the mPFC is thought to underlie extinction learning, in which fear extinction recall is associated with increased phosphorylation of mitogen-activated protein kinase (Russo et al., 2019), and the inhibition of MAPK signaling immediately following extinction via an infusion of the MAPK inhibitor PD098059 in the mPFC impaired extinction retention and recall (Hugues et al., 2004). Glutamatergic signaling from the IL to the NAC shell may be a critical circuit in the consolidation of fear extinction learning. The inactivation of metabotropic glutamate receptor 1 in the NAC shell via an infusion of 1-aminoindan-1,5-dicarboxylic acid (AIDA) 5 min before extinction training also impaired extinction recall 24 h after training (Dutta et al., 2021). Notably, an infusion of PD098095 in the basolateral amygdala (BLA) 10 min before extinction training also impaired the retention and recall of fear extinction (Lu et al., 2001).

The PFC becomes involved in drug-seeking behavior during extinction training. The inactivation of both the PL and IL on the first day of extinction training had no influence on responses on the active lever that was previously paired with cocaine delivery (Peters et al., 2008). Following extinction training, inactivation of the PL or its direct target, the NAC core, reduced the reinstatement of cocaine seeking (Peters et al., 2008). Activity of the IL opposes activity of the PL. Stimulation of the IL with α-amino-3-hydroxyl-5-methyl-4-isoxazole-propionic acid (AMPA), a glutamate receptor agonist, also blocked drug-induced reinstatement in male Sprague-Dawley rats that had undergone extinction training, and this reduction correlated with higher neuronal activity in the IL (Peters et al., 2008). Infralimbic cortex activity is also associated with expression of the extinction of responding for alcohol (Marchant et al., 2010) and heroin (Ovari and Leri, 2008). Inhibition of the IL with γ-aminobutyric acid (GABA) receptor agonists or dopamine receptor antagonists exacerbated the reinstatement of drug seeking (Peters et al., 2008). Similar to the reinstatement of extinguished fear responses (Knapska and Maren, 2009) and the cue-induced reinstatement of cocaine seeking, the reinstatement of drug seeking that is triggered by IL inactivation relies on activity of the PL and BLA (Peters et al., 2008; Sun and Rebec, 2005). Likewise, inhibition of the PL blocked the stress (footshock)-induced reinstatement of cocaine-seeking behavior (Capriles et al., 2003; McFarland et al., 2004). Although inhibition of the IL (via tetrodotoxin) reportedly had no significant influence on the footshock-induced reinstatement of cocaine seeking (Capriles et al., 2003), the literature suggests that stimulating the IL may promote the inhibition of fear or drug seeking that is learned through associative processes and may serve as a therapeutic target to reduce the stress-induced reinstatement of drug seeking.

### 3.2 Infralimbic cortex and alcohol-seeking behavior

Diverse brain structures have been described to play a role in alcohol-seeking and -taking behavior. The decision to engage in disproportionate alcohol drinking may, in fact, be controlled by the mPFC, which has long been described as a crucial player in executive brain function in both primates and rodents (Heidbreder and Groenewegen, 2003; Wood and Grafman, 2003). Although the PL is believed to underlie the implementation of executive behaviors, the IL appears to play an important role in response inhibition (Moorman and Aston-Jones, 2015; Moorman et al., 2015). The initiation and promotion of drug-seeking behavior has been shown to be a function of PL activation, whereas inhibition of this behavior has been observed following IL activation (Moorman et al., 2015; Van den Oever et al., 2010). This has been observed preclinically, in which long-term exposure exerted a greater influence on cell excitability and transmission in the IL, which may emphasize the possibility that the IL may exert more pronounced control over alcohol-seeking behavior (Pleil et al., 2015). Evidence suggests inhibitory control of the IL over exacerbated alcohol seeking, a function that was disrupted in Male Wistar rats that underwent alcohol dependence induction (Meinhardt et al., 2013). Consistent with this observation is another study in which male Wistar rats that were given access to a two-bottle choice paradigm exhibited the enhanced activation of CRF and GABAergic neurons in the mPFC during periods of abstinence, and working memory deficits were directly associated with higher alcohol intake during acute abstinence (George et al., 2012). Thus, hypothetically, alcohol use may damage the mPFC, particularly the IL, via its immediate pharmacological actions, but it may also lead to long-term pathophysiology, which may highlight the transition from controlled alcohol drinking to the loss of control of alcohol drinking that is prominent in AUD.

Nevertheless, the exact mechanisms that underlie how alcohol exposure results in mPFC deficits and consequently the loss of control over alcohol consumption remain unknown. Alcohol seeking and taking are perceived as prototypical examples of associative learning, in which alcohol itself may act as a positive reinforcer, and alcohol-associated cues may drive further seeking behavior (Martin-Fardon and Weiss, 2013). Interestingly, associative learning is thought to be specifically determined by local neuronal ensemble interactions, in which the most prominent characteristics of these networks include spatiotemporal patterns of ensemble activation that derive from defined sets of neurons (Buzsaki, 2010; Wallace and Kerr, 2010). Cue-induced alcohol- and saccharin-seeking behavior activated neuronal ensembles in the IL of male Wistar rats that largely overlapped, and these ensembles displayed similarities in both size and organization (Pfarr et al., 2018). On the other hand, operant learning, particularly reward-predictive stimuli, increased the excitability of neuronal ensembles in the PL, while at the same time, decreasing the excitability of non-ensemble neurons (Whitaker et al., 2017). Both the IL and the PL displayed activation signals that were related to “going” during reward seeking and “stopping” during extinction training conditions (Moorman and Aston-Jones, 2015). Similarly, in a conditioned reinstatement model of relapse, the presentation of a previously associated olfactory cue (S^+^) reinstated previously extinguished operant responding for alcohol, with both the PL and IL displaying significantly higher c-*fos* expression (Dayas et al., 2007). Additionally, distinct neuronal ensembles within the ventral mPFC underlie reward and extinction memories, and thus the possibility exists that both may promote and inhibit reward-seeking behavior (Warren et al., 2016). In a context-induced reinstatement approach (i.e., an ABA paradigm), reversible inactivation of the PL attenuated the context-induced reinstatement of beer seeking in male Long-Evans rats and did not produce any effect of the latency to initiate responding. This IL inactivation, however, augmented the reacquisition of beer seeking. In contrast, inactivation of the IL had no effect on reinstatement, reacquisition, or extinction expression. Interestingly, inactivation of the IL selectively increased response latencies when the rats were tested in the extinction context (Willcocks and McNally, 2013). Considering these data, a major role of the mPFC appears to be its use of contextual information to guide responding based on a benefit *vs.* cost approach, whereby the PL plays a significant role in the retrieval of alcohol-seeking contingency information, whereas the IL is more involved in the actual use of contextual information.

Throughout most areas of the cerebral cortex, particularly within the mPFC, scattered CRF-immunoreactive cells can be found. Interestingly, earlier cell morphology approaches identified these cells as CRF-expressing interneurons (See Fig. 1; Swanson et al., 1983). Although the exact role of this neural ensemble remains a topic of investigation in the field of neuroscience, one study recently identified these neurons as GABAergic inhibitory interneurons (P. Chen et al., 2020). Notably, immunohistochemical evidence has shown that the mPFC, include the PL and IL, receives inputs from the hypothalamus (See Fig. 1; Date et al., 1999). An anterograde anatomical tracer study showed that the IL also projects to dorsomedial, lateral, perifornical, posterior, and supramammillary nuclei of the HYP (See Fig. 1; Vertes, 2004). These projections are understood to stem from pyramidal cells in layer 5 of the IL. These cells are directly regulated by GABA transmission, oversee regional outputs, and contribute to the production of local oscillatory activity (Buzsaki and Wang, 2012; Gabbott et al., 2005; Guyon et al., 2021). This suggests that the disruption of inputs to the IL from the HYP and disruptions of projections from the IL back to the HYP, which may be induced by alcohol dependence, may participate in long-term dysregulation of the mPFC that is prominent in alcohol-dependent individuals. This dysregulation could subsequently be a key factor in compulsive alcohol-seeking behavior, which is intensified during periods of stress.

## 4 Orexin

The excitatory neuropeptide OX (also known as hypocretin), derived from prepro-OX, is synthesized exclusively in cells of the HYP (de Lecea et al., 1998; Sakurai et al., 1998) that project to numerous regions that are commonly associated with stress responses and drug dependence, including the VTA, NAC, CeA, bed nucleus of the stria terminalis (BNST), and mPFC (See Fig. 1; Peyron et al., 1998). Two OX receptors, OX_1_ and OX_2_, have been identified. OX_1_ receptors bind to orexin A (OXA) with higher affinity than to orexin B (OXB), whereas OX_2_ receptors bind to OXA and OXB with similar affinities (Sakurai et al., 1998). Orexin is a promoter of arousal that helps regulate sleep/wake (Chemelli et al., 1999; Hoyer and Jacobson, 2013; Inutsuka and Yamanaka, 2013; Krystal et al., 2013), stress (Berridge et al., 2010; Sargin, 2019), and rewarding effects of food and drugs of abuse (Harris et al., 2005; Martin-Fardon and Boutrel, 2012; Matzeu and Martin-Fardon, 2020; Moorman and Aston-Jones, 2009; Thannickal et al., 2018).

### 4.1 Role of orexin in fear/stress responses

Initial studies after the discovery of OX (Sakurai et al., 1998) demonstrated that fasting upregulates HYP *Ox* mRNA. Acute immobilization and restraint stress also increase *Ox* mRNA levels in male Wistar rats (Ida et al., 2000) and OX neuron activity (Fos expression) in male Wistar rats (James et al., 2014) and in Male C57BL/6 mice (Tung et al., 2016). In male patients who had suffered from depression, OX immunoreactivity and *HCRTR2* mRNA expression increased in the anterior cingulate cortex compared with control males (Lu et al., 2017). In preclinical studies, female Sprague-Dawley rats that were exposed to chronic unpredictable stress exhibited higher *Hcrtr1* mRNA in the frontal cortex compared with control females (Lu et al., 2017). Notably, footshock exposure increased *Ox* mRNA expression, which lasted up to 14 days post-shock, and this increase in mRNA positively correlated with the amount of freezing behavior when the male Sprague-Dawley rats were reintroduced to the footshock context 1–2 days later (X. Chen et al., 2014). Moreover, OX was shown to promote the influence of stress on behavior (Dong et al., 2015; Li et al., 2010; Martin-Fardon and Boutrel, 2012), and OXA infusions in the lateral ventricle elevated intracranial self-stimulation thresholds, which is associated with negative affective states (Boutrel et al., 2005). Orexin and CRF neurons in the HYP provide reciprocal excitatory feedback to one another (for review, see (Sargin, 2019), and OX interactions with CRF are partially responsible for the role of OX in stress responses (Sargin, 2019). Consistent with excitatory feedback that OX provides to hypothalamic CRF neurons, OX administration also increases plasma ACTH and corticosterone levels (Kuru et al., 2000). The stimulation of OX neurons reduced the time spent in a social interaction zone in male Sprague-Dawley rats (Heydendael et al., 2014). Microinfusions of OXA or OXB in the paraventricular nucleus of the thalamus (PVT) had an anxiogenic effect on behavior in the elevated plus maze (Li et al., 2010). In male C57BL/6NCrj mice and male Wisatr rats, an intracerebroventricular injection of OXA exerted an anxiogenic effect on behavior in the light-dark box (Suzuki et al., 2005). In contrast, OX receptor antagonism reduced the latency to enter a social interaction zone in male Sprague-Dawley rats (Dong et al., 2015) and attenuated anxiogenic effects of footshock stress exposure (X. Chen et al., 2014; Li et al., 2010) or a cat (i.e., predator) odor (Staples and Cornish, 2014). Likewise, peripheral administration, intracerebroventricular administration, or an intra-locus coeruleus microinjection of the OX_1_ receptor antagonist *N*-(2-methyl-6-benzoxazolyl)-*N*′-1,5-naphthyridin-4-yl urea (SB334867) before or immediately after fear conditioning impaired fear memory 24 h later in both male C57BL/6J mice and male Sprague-Dawley rats (Flores et al., 2014; Sears et al., 2013). Notably, the role of OX in stress and fear responses may increase in highly salient stress paradigms (Flores et al., 2014; Sargin, 2019).

### 4.2 Role of orexin in alcohol-seeking behavior

Orexin neurons are activated by stimuli that are associated with food and drugs of abuse, such as cocaine, morphine, and alcohol (Dayas et al., 2008; Harris et al., 2005; Jupp et al., 2011), and OX mediates reward by acting directly on dopaminergic neurons in the VTA (Espana et al., 2010; Korotkova et al., 2003; Tung et al., 2016) and potentiating *N*-methyl-D-aspartate (NMDA) receptor-mediated neurotransmission that is critical for drug-induced synaptic plasticity (Borgland et al., 2006). Additionally, OX acts in a positive feedback relationship with alcohol, in which alcohol intake increases OX expression to promote continued alcohol intake (Barson and Leibowitz, 2016). An OXA infusion in the paraventricular nucleus of the HYP or lateral HYP increased alcohol intake (Schneider et al., 2007). In contrast, systemic administration of the OX_1_ receptor antagonist SB334867 reduced alcohol preference in male alcohol-preferring (iP) and high-alcohol preferring outbred male Sprague-Dawley rats (Lawrence et al., 2006; Moorman and Aston-Jones, 2009) and attenuated the conditioned reinstatement of alcohol-seeking behavior in male Wistar rats (Martin-Fardon and Weiss, 2014). Similarly, the antagonism of OX_2_ receptors with JNJ-10397049 (s.c.) reduced alcohol self-administration and the acquisition, expression, and reinstatement of alcohol-induced conditioned place preference in male DBA/2 mice (Shoblock et al., 2011). Adolescent male Wistar rats that were exposed to intermittent alcohol exposure exhibited an increase in OXA immunoreactivity (Amodeo et al., 2020), indicating that drug exposure influences OX expression. In clinical studies, during early withdrawal, individuals who were diagnosed with AUD had high blood levels of OX (Ziolkowski et al., 2016), which correlated with global distress indices (von der Goltz et al., 2011).

The role of OX and influence of OX system manipulations on reward-seeking behavior are exacerbated when salience of the reward increases, such as in the case of drugs (Borgland et al., 2009; Cason and Aston-Jones, 2013; Flores-Ramirez et al., 2022a, Flores-Ramirez et al., 2022b; Martin-Fardon and Boutrel, 2012; Zink et al., 2018). For example, OX_1_ receptor antagonism selectively reduced responding for alcohol but not sucrose under a progressive-ratio schedule of reinforcement in male iP rats (Jupp et al., 2011) and reduced the conditioned reinstatement of alcohol- but not SuperSac- (a palatable sweet solution) seeking behavior (Martin-Fardon and Weiss, 2014) in male Wistar rats. The antagonism of OX_2_ receptors also selectively reduced the self-administration of alcohol but not saccharin in male Wistar rats (Shoblock et al., 2011). Furthermore, OX_2_ receptor antagonism in the nucleus accumbens core, but not shell, with TCSOX229 decreased alcohol self-administration, but not cue-induced seeking in Indiana alcohol-preferring male rats (R. M. Brown et al., 2013). The role of OX in determining drug-seeking behavior may thus also be enhanced during stress or when levels of arousal are high (Berridge et al., 2010). For example, OX cells display greater activation during the stress (yohimbine)-induced reinstatement of alcohol-seeking behavior (Kastman et al., 2016; Richards et al., 2008), and blocking OX activity attenuated the stress-induced reinstatement of alcohol-seeking behavior in both male and female Wistar rats (Flores-Ramirez et al., 2022a, Flores-Ramirez et al., 2022b; Matzeu and Martin-Fardon, 2020). Recently, a case study showed that an individual with comorbid AUD and insomnia, who was treated with the dual orexin receptor antagonist suvorexant (Belsomra®), an FDA-approved insomnia treatment led to improvements in alcohol cravings, overall physical and psychological health, as well as sleep quality (Campbell et al., 2024).

## 5 Dynorphin

The peptide DYN (Goldstein et al., 1979), derived from pro-DYN, is the endogenous ligand for and preferentially binds to κ-opioid receptors (KOPs; (Chavkin et al., 1982), although DYN binds to all three opioid receptor subtypes (Schwarzer, 2009). The precursor pro-DYN can be processed to DYNA(1–17), DYNA(1–8), and DYNB(1–29) (Fallon and Leslie, 1986). Unlike OX, DYN is also synthesized in areas outside the HYP (Bali et al., 2014; Watson et al., 1982). However, like OX, actions of DYN are associated with various physiological processes and reinforcing effects of drugs of abuse (Bruchas et al., 2009; Wee and Koob, 2010). In human brains, high levels of *PDYN* mRNA expression are found in the striatum (STR), NAC, amygdala, and mPFC (Hurd, 1996). Interestingly, DYN is found within mesolimbic projections from the NAC to the VTA (Shippenberg et al., 2001), a circuit whose role is well characterized in addiction research. Opposing excitatory effects of OX, DYN inhibits receiving synapses (Muschamp et al., 2014; Reeves et al., 2022). As such, KOP activation decreases mesolimbic dopamine by decreasing dopamine release and increasing dopamine uptake (Shippenberg et al., 2001).

### 5.1 Role of dynorphin in fear/stress responses

Stress increases DYN expression in various brain areas. Restraint/immobilization and learned helplessness stress increase *Pdyn* mRNA in the HYP (Palkovits, 2000), the region where OX is exclusively synthesized (de Lecea et al., 1998), as well as the hippocampus and NAC (Bali et al., 2014; Lucas et al., 2011; Shirayama et al., 2004). In preclinical studies, KOP agonist elicited conditioned place aversion (Mucha and Herz, 1985) and depressive-like effects in the forced swim test in male Sprague-Dawley rats (Carlezon et al., 2006). Additionally, the activation of KOPs in the BLA by CRF mediated anxiety-like behavior in the elevated plus maze in male C57BI/6 mice (Bruchas et al., 2009). Repeated KOP activation by forced swim stress also dysregulated the postsynaptic activation of G-protein-gated inwardly rectifying potassium channels, regulating the excitability of serotonergic neurons in the dorsal raphe nucleus through a p38α MAPK-dependent mechanism (Lemos et al., 2012). Notably, KOP agonists also produce aversion and dysphoria in drug-naïve male Sprague-Dawley rats (Mucha and Herz, 1985) and humans (Pfeiffer et al., 1986), respectively. In contrast, KOP antagonism increases anxiolytic effects in the elevated plus maze in male Sprague-Dawley rats (Knoll et al., 2007), produces anti-depressant effects in learned helplessness tasks (Shirayama et al., 2004), and induces analgesic and antinociceptive responses in the repeated forced swim test in male C57Bl/6 mice (McLaughlin et al., 2003) and tail pinch test following restraint stress in male ICR mice (Suh et al., 2000).

### 5.2 Role of dynorphin in alcohol-seeking behavior

Like stress, drug use also influences DYN expression. Although DYN opposes actions of OX by decreasing alcohol intake, alcohol intake increases the expression of both OX and DYN (Barson and Leibowitz, 2016). The systemic administration of KOP agonists (e.g., salvinorin A) has been shown to elevate intracranial self-stimulation thresholds (Carlezon et al., 2006), opposing behavioral effects of repeated drug use (Shippenberg et al., 2001) in part by decreasing dopaminergic activity in the NAC (Maisonneuve et al., 1994) and PFC (Margolis et al., 2006). Alterations of dopamine activity in the NAC and mPFC that are associated with cocaine use are blocked by the co-administration of KOP agonists (Shippenberg et al., 2001), and the intracerebroventricular administration of DYN-A had similar effects on dopamine activity in response to heroin in male Long-Evans rats (Xi et al., 1998). Acute KOP agonism by the systemic administration of salvinorin A or (5R)-(5α,7α,8β)-N-methyl-N-[7-(1-pyrrolidinyl)-1-oxaspiro[4,5]dec-8-yl]-4-benzofuranacetamidemonohydrochloride (CI-977 also known as enadoline) likewise lowered the rewarding effect of intracranial self-stimulation in male Wistar and Sprague-Dawley rats (Holter et al., 2000; Potter et al., 2011). Additionally, systemic 2-(3,4-dichlorophenyl)-N-methyl-N-[(1R,2R)-2-pyrrolidin-1-ylcyclohexyl]acetamide (U50,488H) or bremazocine administration decreased alcohol drinking in male Lewis and Wistar rats (Lindholm et al., 2001; Nestby et al., 1999). In high-alcohol-drinking male Lewis rats, KOP antagonism with nor-binaltorphimine (norBNI) increased free-access alcohol drinking (Mitchell et al., 2005). However, in alcohol-dependent male Wistar rats, norBNI attenuated withdrawal-induced increases in drinking (Walker and Koob, 2008). Such results suggest that drug-induced upregulation of the DYN/KOP system underlies negative affective states that are associated with drug withdrawal that likely promote drug seeking well into drug abstinence (Koob, 2008). In drug dependence, KOP activation may increase stress and dysphoria to elicit the reinstatement of drug-seeking behavior (Wee and Koob, 2010). Notably, repeated cocaine administration increases *Pdyn* mRNA expression and decreases KOP expression in the NAC and STR up to 48 h after cocaine exposure in male Wistar rats (Turchan et al., 1998). Withdrawal from alcohol (Lindholm et al., 2000) was also shown to be associated with an increase in *Pdyn* mRNA expression in male Sprague-Dawley rats. KOP antagonism by systemic norBNI administration attenuated increases in stress responsiveness that are associated with alcohol withdrawal in male Wistar rats (Gillett et al., 2013). Chronic administration of the KOP agonist CI-977 also potentiated relapse-like alcohol intake in male Wistar rats (Holter et al., 2000). As such, KOP antagonism during withdrawal may relieve withdrawal- and stress-related motivation to reinstate drug-seeking behavior (Wee and Koob, 2010). Likewise, the stress-induced reinstatement of cocaine-induced conditioned place preference is inhibited by the genetic deletion of KOPs or pro-DYN in male C57BL/6J mice (Redila and Chavkin, 2008). Treatment with KOP antagonists also inhibited the stress (intermittent footshock or forced swim)-induced reinstatement of cocaine-induced conditioned place preference and cocaine-seeking behavior in male Long-Evans hooded rats and male C57BL/J mice (Beardsley et al., 2005; Redila and Chavkin, 2008). norBNI pretreatment before the forced swim test or *Pdyn* knockout in C57Bl/6J mice also blocked potentiated cocaine-induce conditioned place preference that was associated with prior exposure to stress (McLaughlin et al., 2003). Still to be determined is whether KOP antagonism can also prevent the stress-induced reinstatement of alcohol-seeking behavior, potentially by reducing the impact of stress mechanisms and/or responses.

## 6 Orexin and dynorphin interactions

Orexin release promotes dopamine-driven drug seeking, but the administration of an OX_2_ receptor antagonist (JNJ-10397049) effectively reduced alcohol self-administration in male Wistar rats and conditioned place preference in male DBA/2 mice without significantly reducing NAC dopamine levels (Shoblock et al., 2011), suggesting the involvement of other systems. Importantly, nearly all (94%) hypothalamic cells that express *Ox* mRNA also express *Pdyn* mRNA (Chou et al., 2001; Li & van den Pol, 2006). Furthermore, OX and DYN are located within the same synaptic vesicles and are co-released (Li & van den Pol, 2006; Muschamp et al., 2014), indicating that OX and DYN likely interact in brain regions to which OX neurons project, including the IL (see Fig. 1). A study by Muschamp et al. (2014) found that OX_1_ receptor antagonism with systemic SB334867 administration or an SB334867 microinfusion in the VTA in male C57BL/6J mice elevated intracranial self-stimulation thresholds reinforced by lateral HYP stimulation. Interestingly, KOP antagonism with systemic or intra-VTA norBNI alone did not have a reciprocal influence on intracranial self-stimulation thresholds. However, pretreatment with norBNI prevented the SB334867-induced elevation of intracranial self-stimulation thresholds, indicating that these peptides interact within the VTA. Most (65.4%) of dopaminergic neurons in the VTA in mouse brain slices responded to both OX and DYN and displayed no net effect on firing rates when exposed to both peptides, whereas some responded preferentially to either OX (16.9%) or DYN (7.7%; Muschamp et al., 2014). On the other hand, exogenous application of OX and DYN to the lateral HYP results in contrasting effects on neuronal firing in mice (Li & van den Pol, 2006). Furthermore, optical stimulation of lateral HYP OX/DYN in male and female OX-Cre mice resulted in neuromodulation of dopamine cell firing in the VTA, and this increase in firing was inhibited by a OX_1_ receptor antagonist. The decrease in firing was inhibited by a KOR antagonist, and when both peptide receptors were blocked, OX and DYN stimulation in the lateral HYP did not alter firing, which may indicate that the changes in firing are mediated by peptide release into the VTA (Mohammadkhani et al., 2024). In this context, it could be argued that one peptide's effects over the other is likely influenced by an array of factors to include the relative levels of each peptide within the synaptic vesicles, their concentration and prevalence in the extracellular environment, their exact receptor distribution among different neuronal populations, and the interactions between their target receptors and their intracellular signaling pathways in postsynaptic cells (Muschamp et al., 2014), and that this influence may be differential in a brain region-dependent manner.

With regard to drug-seeking behavior, the balanced opposing effects of OX and DYN in the VTA also appear to occur in the PVT. A study by Matzeu et al. (2018) found that the administration of OX alone in the PVT reinstated cocaine-seeking behavior, and the co-administration of OX with DYN blocked the OX-induced reinstatement of cocaine-seeking behavior in male Wistar rats that had a history of long access (LgA, 6 h/day) to cocaine by attenuating OX-induced glutamate release. Importantly, the coadministration of OX with DYN in the PVT did not reduce the OX-induced seeking of a highly palatable sweet solution (Matzeu et al., 2018), suggesting that the influence of OX and DYN interactions in the PVT and potentially other regions is altered by excessive drug consumption and, in turn, drug dependence. Embryonic alcohol exposure of zebrafish has been reported to reduce the proportion of HYP OX neurons that co-express DYN (Yasmin et al., 2023), indicating that drug exposure may augment the ability of OX to promote drug seeking over the ability of DYN to reduce drug seeking. Although the responsivity of IL cells to co-release OX and DYN has not yet been tested directly, evidence from other regions suggests that these two peptides oppose one another's actions, particularly when originating from the HYP. However, the ability of DYN to oppose OX's ability to promote drug seeking may be reduced in alcohol dependence, especially under conditions of acute stress.

## 7 Orexin and corticotropin-releasing factor interaction

Several neuropeptides have been suggested to interact with CRF, either facilitating or inhibiting the recurrence of drug-seeking behavior (Shalev et al., 2010). Functional and behavioral approaches have underscored the significant role of the OX system in neurobehavioral and motivational consequences of drug abuse, including alcohol (Borgland et al., 2006; Harris et al., 2005; Thompson and Borgland, 2011). Interestingly, the activation of OX cells has been observed as a function of exposure to stress, and OX itself has been postulated to play a modulating role in stress responsivity (Giardino and de Lecea, 2014; Ida et al., 2000). Neuroanatomical experimental approaches have revealed that CRF terminals that originate in the PFC make direct contact with OX cells in the HYP that express CRF_1_ and CRF_2_ receptors, and neurons that express CRF receive connections from the OX system (Winsky-Sommerer et al., 2004). Additionally, an important observation was that the activity of OX neurons in the HYP, when studied *ex vivo* in brain slices from male CRF_1_ knockout and wildtype mice, was directly and proportionally stimulated by CRF, providing compelling evidence that these two systems have a direct interaction with each other within this limbic system brain structure (Winsky-Sommerer et al., 2004). Corroborating these anatomical studies is behavioral evidence of an interaction between OX and CRF. For example, intracerebroventricular OX administration in male Wistar rats led to an increase in anxiety-like behavior and dose-dependently reinstated operant cocaine seeking without altering intake, and it dramatically elevated intracranial self-stimulation thresholds, which was mediated by CRF (Boutrel et al., 2005; Hata et al., 2011; Suzuki et al., 2005). Overall, these finding suggest that the modulation of CRF neurons by OX plays an important role in sustaining and prolonging negative emotional states that are characteristic of alcohol dependence and may, in fact, increase the vulnerability to relapse in dependent subjects.

Research has also shown that the interaction between OX and CRF is reciprocal, given that CRF stimulates OX neurons, and these neurons activate CRF-dependent transcription when exposed to different stressors (Winsky-Sommerer et al., 2004). Thus, theoretically, when an individual abruptly stops using alcohol after a prolonged period of time, this triggers a state of heightened alertness that resembles stress, and this withdrawal state, in turn, enhances the transcriptional activity of OX neurons in the lateral HYP (Georgescu et al., 2003). Importantly, the drug withdrawal-induced activation of several brain structures decreased after systemic OX_1_ receptor antagonism (Laorden et al., 2012; Plaza-Zabala et al., 2012). Moreover, a study (Boutrel et al., 2005) found that intracerebroventricular OXA administration in male Wistar rats induced cocaine-seeking behavior, and this specific effect depended on CRF. The same study (Boutrel et al., 2005) also found that inhibiting noradrenergic and CRF systems prevented drug-seeking behavior that was induced by OX, which may imply that OXA may trigger drug-seeking behavior by inducing a “stress-like” state via these systems. Further validating this interpretation, this group also found that intraperitoneal SB334867 administration prevented the footshock stress-induced reinstatement of previously extinguished cocaine-seeking behavior (Boutrel et al., 2005). Interestingly, a separate study reported that systemic SB334867 administration prevented the stress (yohimbine)-induced reinstatement of alcohol-seeking behavior, without affecting locomotor activity in male Long-Evans rats (Richards et al., 2008). Altogether, these results show that the functional interaction between OX and CRF neurons may play an essential role in persistent, negative emotional states that are associated with drug dependence and, more importantly, that OX receptor antagonism can ameliorate CRF-dependent stress responsivity.

Research indicates that this particular interaction between CRF and OX exists within the IL, given that the IL receives OX projections from the HYP, and *Hcrtr1* and *Hcrtr2* mRNA within this brain structure has been detected (Date et al., 1999; Marcus et al., 2001). Recently, the behavioral implication of a CRF and OX transmission interaction within the IL was tested by our group (Flores-Ramirez et al., 2022a, Flores-Ramirez et al., 2022b). Male Wistar rats were trained to self-administer 10% alcohol over 3 weeks. Afterward, the rats were subjected to 2 weeks of extinction training. Twenty-four hours after the last extinction session, the rats were given bilateral intra-IL injections of the CRF_1_ receptor antagonist CP154,526, the dual OX receptor antagonist TCS1102, or a combination of both. The rats were then tested for the reinstatement of footshock stress-induced alcohol-seeking behavior. CP154,526 significantly inhibited reinstatement, whereas TCS1102 did not have this effect. Interestingly, when TCS1102 and CP154,526 were co-administered, it negated the effect of CP154,526 alone. Notably, footshock stress led to a significant increase in the expression of *Crhr1* and *Hcrtr2* mRNA in the IL. These findings highlight a functional interaction between OX and CRF receptor signaling in the rodent mPFC during the reinstatement of alcohol-seeking behavior that is precipitated by stress.

## 8 Where to go from here?

Despite its complexities, the role of the mPFC, particularly the IL, in the compulsive and relapsing nature of AUD is a promising subject for further study. Of the many gaps in the literature yet to bridge, perhaps the greatest one is the fact that, unfortunately, most of the studies discussed in this review were conducted using male animals as model systems. Of course, if we are to advance our understanding of brain mechanisms, and ultimately improve health outcomes for *everyone*, future research that aims to uncover the sex-differences that underlie the neurobehavioral ontogeny and pervasiveness of AUD is imperative. To this end, it has been shown that men report greater alcohol craving in response to cues than women (Wang et al., 2019), that women displayed greater performance than men to obtain alcohol using a post-abstinence paradigm (Plawecki et al., 2018), alcohol-seeking behavior in response to alcohol availability is greater in female BDNF heterozygous rats, and female C57BL/6J mice display greater aversion-resistant alcohol drinking (Fulenwider et al., 2019; Hogarth, Jaehne, van den Buuse and Djouma, 2018). Similarly, sex-differences in CRF, OX, and DYN systems and their contribution to psychiatric disorders have been uncovered (Bangasser, 2013; Karkhanis and Al-Hasani, 2020; Zhang et al., 2024). Of special importance will be whether the IL, or its human analogue, the ventromedial PFC shows sexually dimorphic functioning, particularly as it pertains to the peptides discussed, and in its relationship to AUD. Understanding these issues will be crucial, especially for their implications in pharmacotherapy development.

It would be an understatement to say that the neural networks in the brain, particularly those found in the IL, are complicated. There are a myriad of neuronal subtypes within this subregion of the mPFC whose neural circuit functions remain unknown. In this context, more contemporary tools such as optogenetics (Deisseroth, 2015) and chemogenetics (Roth, 2016), have risen to the forefront of neuroscience to help us answer these complex questions. These tools have revolutionized basic research by allowing investigators to uncover altering physiological processes in target cells, as well as disrupt these processes to induce pathological conditions. Of course, this mechanistic approach is valuable for testing hypotheses about the root causes of AUD and for studying its progression as it affects specific populations of cells, particularly in the IL. Much of the research presented in this review employed behavioral pharmacology techniques, which are limited by the pharmacokinetics of the ligand or compound utilized. Thus, the development of these biosynthetic approaches posits a unique and exciting opportunity for the field to further uncover the functional interaction between CRF, OX, and DYN within the IL, especially as it relates to exacerbated alcohol-seeking behavior.

Of course, the final frontier that remains is bridging the translational gap and develop medications that *actually* go from the bench to the clinic. In this tenet, pharmacotherapeutics that target the systems herein described have been successful in preclinical models of AUD but have failed to display a clinical therapeutic value. For example, Verucerfront (NBI-77860/GSK561679A), a potent CRF_1_ receptor antagonist failed to show efficacy in reducing alcohol craving and responsivity to alcohol- and stress-related imagery, as well as resulted in an overall increase in anxiety associated with the New Trier social stress test of public speaking in women (Schwandt et al., 2016). Similarly, Pexacerfont (BMS-562,086) also failed to alleviate craving, emotional responsivity, and/or anxiety (Kwako et al., 2015). Chronic treatment with LY2456302, a KOP receptor antagonist, did not reduce measures of depression or cocaine craving in a stress-minimized inpatient setting (Reed et al., 2018). Additionally, administration of another KOP receptor antagonist, CERC-501 (also known as LY2456301), did not affect cigarette smoking, craving, or nicotine withdrawal in a randomized, double-blind, placebo-controlled study in a human laboratory model of smoking behavior (Jones et al., 2020). Moreover, even though the clinical effects of suvorexant were reported to improve alcohol cravings, overall physical and psychological health, as well as sleep quality (Campbell et al., 2024), it was shown to enhance the reinforcing effects of intravenous cocaine in humans (i.e., increased self-administration of 10 mg/70 kg cocaine). While these mostly null effects may appear grim, on the surface, we would argue that they still represent an exciting avenue of research for the field, as they may demonstrate the sobering fact that the “silver bullet” may not exist. As such, the complexity of a psychiatric disorder such as AUD may warrant for a multi-target treatment approach that considers the functional interactions between different reward/antireward systems, namely CRF, OX, and DYN and particularly in brain regions that underlie executive function, long believed to be involved in the vulnerability to relapse that is precipitated by stress.

## 9 Conclusion

Overall, the IL emerges as a critical nexus for addressing the multifaceted challenges outlined in this review, underscoring its pivotal role in the etiology and persistence of AUD. Given the intricate interplay between CRF, OX, and DYN within the IL, this region is uniquely positioned to mediate compulsive alcohol-seeking behaviors, and resulting vulnerability to stress-induced relapse, that characterize AUD. The urgency to focus research efforts on the IL is heightened by its demonstrated influence on both the extinction and reinstatement of conditioned fear and drug-seeking responses, which are central to understanding and mitigating relapse. The IL's ability to integrate and modulate stress and reward pathways through its dense CRF and OX receptor populations makes it a prime target for developing multi-faceted therapeutic interventions that may be both safe and effective. Furthermore, the co-release of OX and DYN within the IL, and their opposing effects on neuronal excitability, highlight the potential for novel pharmacological strategies that can finely tune these interactions to curb compulsive behaviors. As this review elucidates, disruptions in IL function due to chronic alcohol exposure underscore the transition from controlled to compulsive drinking, emphasizing the need for targeted interventions that could restore IL integrity and functionality. The methodical evidence presented here calls for a future concentrated research focus on the IL to unravel its complex neurocircuitry. Ultimately, addressing the IL's role in stress and reward processing is not just a promising avenue for basic science but an urgent imperative to combat the pervasive and relapsing nature of AUD.

## CRediT authorship contribution statement

**Francisco J. Flores-Ramirez:** Writing – review & editing, Writing – original draft, Conceptualization. **Jessica M. Illenberger:** Writing – review & editing, Writing – original draft, Conceptualization. **Rémi Martin-Fardon:** Writing – review & editing, Writing – original draft, Funding acquisition, Conceptualization.

## Declaration of competing interest

None.
